# Oral-Health-Related Quality of Life as Measured with the Child-OIDP Index and Oral Health Status in Spanish Adolescents

**DOI:** 10.3390/ijerph191912450

**Published:** 2022-09-29

**Authors:** María Paloma Alvarez-Azaustre, Rossana Greco, Carmen Llena

**Affiliations:** 1 Department of Dentistry, Faculty of Biomedical and Health Sciences, Universidad Europea de Valencia, Paseo de la Alameda 7, 46010 Valencia, Spain; 2 Department of Stomatology, Faculty of Medicine and Dentistry, Universitat de València, c/Gascó Oliag 1, 46010 Valencia, Spain

**Keywords:** oral health, quality of life, dental caries, surveys and questionnaires, adolescent

## Abstract

Background: Our aim was to analyze the relationship between OHRQoL (Oral-Health-Related Quality of Life) assessed by the C-OIDP (Child-Oral Impacts on Daily Performances) indexand oral health status in 13-15 years old Spanish population. Methods: A cross-sectional study was designed in a random sample of 337 adolescents. The C-OIDP and an oral health perception questionnaire were applied. ICDAS II (International Caries Detection and Assessment System) classification was used for assessing caries status, CPI (Community Periodontal Index) for periodontal status and discrepancy in the three planes for occlusal evaluation. Statistical analysis included Mann–Whitney, Kruskal–Wallis and Spearman correlation tests. Results: The prevalence of impact was 48.1%. The most frequently affected dimension was eating (21.4%). The overall mean C-OIDP index was 3.28. Higher DMFT (Decayed Missed Filled Tooth) was significantly associated with C-OIDP index (*p* = 0.03). Participants with CPI > 0 showed an impact index almost twice as high as those with CPI = 0 (*p* > 0.05). Significant association was found between quality of life and CPI for women (*p* < 0.01). Only the molar Angle’s class was associated with the impact index (*p* = 0.04). Conclusions: The OHRQoL in Spanish adolescents is good. The DMFT index had an impact on adolescents’ quality of life, showing a greater impact in men than in women. Periodontal status had a greater impact in women. No association between the extent of the caries lesion and the impact index was found.

## 1. Introduction

Scientific progress and community public health efforts have led to a remarkable improvement in the oral health status of the new generations [1]. At the same time, there has been a change in the expectations of patients who not only expect to receive treatment for their oral pathology but also to have their perceived health needs met [2]. In response to these expectations, the need arose to design instruments to measure the subjective aspects of oral health [3], and the oral quality of life questionnaires emerged.

Quality of Life (QoL) is a person’s perception of their position in life, based on their expectations, interests and value system and within their cultural context [4]. Quality of life only has meaning on a personal level and is understood differently in the various cultures [5], which requires adapting the questionnaires to assess the socio-cultural environment in which they are to be applied. From QoL derives Oral-Health-Related Quality of Life (OHRQoL), which is a multidimensional construct that includes the perceived impact of oral health on physical, psychological, functional and social aspects [6].

OHRQoL questionnaires were developed for adults in the last decade of the 20th century, including the Geriatric Oral Health Assessment Index (GOHAI) [7], Oral Health Impact Profile (OHIP) [8], Dental Impact on Daily Living (DIDL) [9] and Oral Impacts on Daily Performances (OIDP) [10].

The implementation of children’s questionnaires became more complex due to the need to adapt to cognitive changes at this stage of development [11,12], and has taken place since the beginning of the 21st century, being used in clinical dentistry, dental epidemiology, and dental health services research [6]. Subjective oral health measures for use with children and adolescents have been defined as a burgeoning field [13]. Among them, the CPQ11-14 (Child Perceptions Questionnaire) [14], the COHIP (Child Oral Health Impact Profile) [15], and the C-OIDP (Child-Oral Impacts on Daily Performances) [16] are the most widely used instruments [6].

The C-OIDP is derived from the same questionnaire in its adult version, with an adaptation of language, question content and recall period. It consists of eight structured questions on eight activities of daily living. It is characterized by capturing the adolescent’s perspective in a more comprehensive way by measuring the frequency and severity of impact [6]. It exists in two forms, generic and condition-specific (CS), with the condition-specific form showing greater discriminative ability when comparing groups with and without caries and with and without malocclusion [17,18].

The C-OIDP was developed in English and then validated in Thailand [16], and has been translated and validated in a variety of socio-cultural contexts, obtaining a rating of 4/6 on the Norman and Streiner scale [19] and showing adequate internal reliability [20]. 

The C-OIDP questionnaire was validated in Spain for use with adolescents by Cortés-Martinicorena et al. in 2010, where a mean C-OIDP score of 2.69 and 3.08 in schoolchildren aged 11–12 and 13–14 years old, respectively, was found, and the association of the C-OIDP index with the self-perception variables was reported [21]. Subsequently Montero et al. analyzed the impact of clinical conditions on the quality of life captured by the Child-OIDP, in a sample of 6–12year-olds, finding that caries and periodontal disease were among the main predictors of the impact on the quality of life [22]. Since then, to our knowledge, no study has analyzed the relation of clinical conditions to oral quality of life, as measured with the C-OIDP index in Spanish adolescents, which is the reason for proposing this research project.

Based on the data of the latest national epidemiological Oral Health Survey conducted in 2020, regarding the oral health status of the adolescent population in Spain, 74% of 12-year-olds and 64.5% of 15-year-olds were caries-free. The DMFT index at 12 years of age shows a decreasing trend from 1.2 in 2010 to 0.58 in 2020, having reached the oral health objectives for caries set by the Spanish Society of Oral Public Health (Sociedad Española de Epidemiología y Salud Pública Oral, SESPO) and by the professional organization [23,24]. The same trend was found at 15 years, with a DMFT index in 2020 of 0.94. The percentage of periodontal healthy schoolchildren in 2020 was 40.8% and 36.7% at the age of 12 and 15 years, respectively. Regarding malocclusion, 43.1% of 12-year-olds and 50.3% of 15-year-olds were free of malocclusion [24].

The relationship between oral quality of life as measured by the C-OIDP index and oral health status has been previously studied in the adolescent population using WHO diagnostic criteria for caries [25]. Several factors, such as previous caries experience, the DMFT (Decayed, Missed, Filled, Tooth) index, caries in primary teeth, canker sores, bleeding gums and malocclusion, have been associated with a lower level of OHRQoL as reported in studies included in a recent systematic review [26].

The ICDAS II (International Caries Detection and Assessment System) classification is more sensitive in differentiating between incipient and extensive caries lesions [27,28], and facilitates the application of therapeutic measures following the criteria of minimum intervention and maximum preservation of dental structures [29,30]. Its use has been recommended by The Brussels Statement on the Future Needs for Caries Epidemiology and Surveillance in Europe (2018) [31].

The aim of this study was to analyze the oral quality of life of adolescents aged 13–15 years in the Valencia Region, using the C-OIDP index, and to relate it to their oral health status using the ICDAS II classification for caries assessment, CPI for periodontal status assessment and malocclusion through analysis in the three planes of space. The null hypothesis was that oral health quality of life evaluated by C-OIDP questionnaire was not correlated with oral health status.

## 2. Materials and Methods

### 2.1. Study Design and Setting

A cross-sectional analytical study was designed following a cluster sampling approach. The clusters were public and private secondary schools in the Valencia region.

The sample selection was made on a population of about 400,000 inhabitants including part of the city of Valencia and other bordering municipalities. The sample included adolescents aged 13–15 years attending the 2nd and 3rd year of compulsory secondary education.

Approval was requested and obtained from the Ethics Committee of Universitat de València (ref.H20190501104101), and authorization was obtained from the schools invited to participate. Parents or guardians of the participants signed an informed consent and the participants accepted to collaborate in the study.

### 2.2. Sample Selection

A list was obtained of the schools that teach 2nd and 3rd year of compulsory secondary education in the geographical area selected, finding that there were 47 schools. A letter was sent to the principals of 25 randomly selected schools, 13 public and 12 private, presenting the study and the collaboration requested, and inviting them to participate. Eight schools accepted to participate, 4 public and 4 private, with the engagement of 50 classrooms. The main reasons for schools not to participate were related to the logistic difficulty of carrying out the proposed project. All of the students in the public and private schools that accepted to participate were enrolled in the study. The calculation of the sample size was based on the impact prevalence data obtained in the previous validation study in Spain [21]. The expected proportion of impact on oral quality of life was estimated to be between 30% and 40%, resulting in a sample size of between 323 and 369 participants. A sample of 337 participants was finally obtained.

### 2.3. Survey Instruments

#### 2.3.1. Oral Health Perception Questionnaire

Oral health perception data included questions on oral health problems and perceived treatment needs [22], and assessment of oral health status and satisfaction with oral health using the Oral Satisfaction Scale (OSS) [32].

#### 2.3.2. Child-OIDP Questionnaire

The questionnaire C-OIDP consists of 8 structured questions asking for the frequency and severity of oral health impact on 8 activities of daily living (eating, talking, brushing teeth, sleeping, emotional state, smiling, schoolwork and playing). By multiplying the frequency and severity of impact in each dimension, intensity of impact is obtained, and referring it to the maximum score that can be obtained, the impact index C-OIDP is calculated, whose value can be from 0 to 100. The higher the index, the worse the oral quality of life (Figure S1) [21].

The formula used is:

$$C-\mathrm{OIDP}=\frac{\sum \left(\mathrm{frequency}\;\times \;\mathrm{severity}\right)}{72}\times 100$$

### 2.4. Clinical Diagnostic Criteria

Clinical exam was conducted by an experienced dentist with more than 10 years of professional experience (MP-AA). Data were written by one assistant dentist (R-G). The experienced dentist performed a training calibration with a calibrated examiner for ICDAS II classification (C-LL) on 25 adolescents of the same age range as the subjects participating in the main study who attended primary care dental consultations. Interexaminer Kappa value was 0.87. Moreover, a pilot study was conducted during this training period, on these 25 subjects, where the C-OIDP questionnaire was administered to determine the face and content validity.

#### 2.4.1. Dental Status

Dental decay status was evaluated using ICDAS II classification [29], where each tooth is assigned 2 digits, the first for the restoration code and the second for the caries code (Table 1) [33]. 

The DMFT index was obtained from the ICDAS codes considering, with respect to the caries extent, codes 4 to 6 [28].

#### 2.4.2. Periodontal Status

Periodontal status was assessed with the Community Periodontal Index (CPI) by examining 6 index teeth in each participant; a detailed description of index teeth may be seen in Results section later on After periodontal probing, each tooth was assigned code 0 if the tooth was healthy, code 1 if there was plaque or bleeding on probing and code 2 if there was tartar or overflowing filling [25].

#### 2.4.3. Occlusal Status

In the participant’s occlusion, the presence of discrepancies in the 3 planes of space was examined. In the axial plane, the anteroposterior molar relationship according to Angle’s classification and the presence of a normal (1–2 mm) or increased overjet were examined. In the frontal plane, the presence of anterior open bite, crowding or diastema of incisors and the existence of a normal (2–3 mm or 1/3 of vestibular surface of the lower central incisor) or increased overbite were explored, and in the transversal plane the existence of posterior crossbite [34].

### 2.5. Field Work

Prior to fieldwork, the examiner (MP-AA) visited all the schools in person and arranged an interview with the management team to explain the objective of the study and the methodology to be followed. They were given an informed consent form to be signed by the participant and the responsible adult, which included an informative part on the objective of the study, the methodology to be used and the benefits expected to be achieved, and another part with the authorization to be signed. The signed forms were collected from the school one week before the visit. 

The fieldwork was conducted from September 2019 to the end of January 2020 and was carried out by the examining dentist (MP-AA) and a fieldwork assistant dentist (R-G). An identification number was assigned to each participant to process the data in an anonymized manner. 

**Figure 1:**
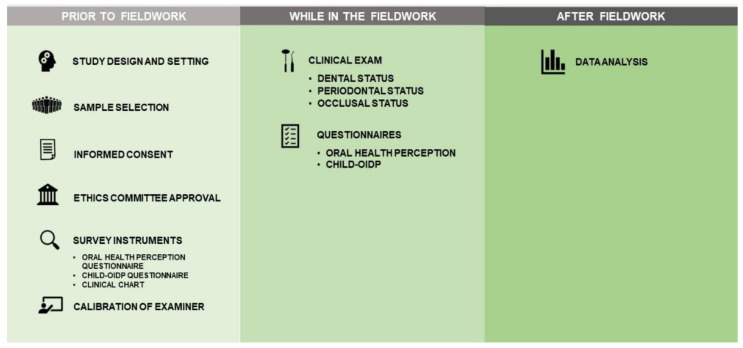
Graphical illustration of the methodology followed in the study.

At each visit, each participant was given a list of 18 pathologies to select the ones they had presented in the last 3 months, and the C-OIDP questionnaire was self-administered simultaneously to all participants in the classroom, followed by the individual oral health perception questionnaire and clinical examination in an adjoining room. An individual dental report was completed for each participant and sent to them via the school administration one week after the visit (Figure 1).

### 2.6. Data Analysis

The psychometric properties of the questionnaire were established in this sample population, including face and content validity, which were tested in the pilot study as well as construct, concurrent and discriminant validity. Internal reliability was tested by using Cronbach’s alpha coefficient, inter-item correlation, corrected item-total correlation and alpha if an item is deleted.

Overall impact prevalence was determined as well as impact prevalence by dimensions, impact index, impact intensity and extent and main causes of impact.

Descriptive analysis of data was followed by association analysis with C-OIDP index used as response variable; as C-OIDP index followed a skewed distribution, non-parametric tests Mann–Whitney U Test, Kruskal–Wallis and Spearman correlation were applied. Data were analyzed using SPSS 28.0 IBM statistical package (Chicago, IL, USA). The statistical significance level was set at *p* < 0.05 in all cases.

## 3. Results

The sample comprised 337 participants, 53.1% females and 46.9% males, with an average age of 13.6 years.

### 3.1. Psychometric Properties of the Child-OIDP 

Face and content validity of the C-OIDP questionnaire were examined and found to be adequate. Face validity refers to the formal design of the questionnaire, which was easily understood by the participants and content validity to the fact that the eight dimensions of the questionnaire reflect the fundamental activities of the adolescent´s life. Construct validity was determined by a statistically significant association between perceived oral health problems, treatment needs and self-assessment of oral health, and the C-OIDP index. Concurrent validity was determined by the significant association between satisfaction with oral health and the C-OIDP index. Additionally, the discriminant validity was determined by finding a significant association between the DMFT index and C-OIDP index.

Regarding internal reliability, inter-item correlation ranged between 0.00 (smiling/schoolwork or smiling/playing) and 0.48 (emotional state/talking). Cronbach´s alpha coefficient was 0.64 and standardized alpha = 0.69. The corrected item-total correlation coefficient was ≥0.40 in most of the dimensions. In two cases, a value < 0.20 was obtained; when deleting those items, no substantial improvement in alpha was seen and it was decided to keep them to allow for comparisons with other studies. Data are shown in Table 2 and Table 3.

### 3.2. Oral Health Perception

The existence of halitosis was reported by 34% of the participants and was referred as a cause of impact by 25% of those reporting it. Subjects with halitosis had higher values of C-OIDP index. Halitosis was more frequent in the morning but had a greater impact if it was perceived in the evening. 

In relation to oral health perception, 68% of participants reported no oral health problems and the main perceived problems were esthetics (9.5%) and gingival bleeding (4.2%). The main perceived treatment needs were orthodontics (25.2%) and teeth cleaning (12.2%). The self-assessment of oral health status showed that 75% of adolescents reported a good state of health, while 82.9% reported being highly satisfied, giving themselves a score of ≥7 out of 10 on the Oral Satisfaction Scale (OSS) in both cases. All parameters of oral health perception analyzed in the study showed a statistically significant association with the impact index, including perception of halitosis (*p* < 0.05).

### 3.3. Impact of Oral Health Measured with Child-OIDP Index

The C-OIDP questionnaire revealed an impact prevalence of 48.1%, where eating was the most frequently affected dimension (21.4%), followed by smiling (19.3%) and emotional state (18.1%), and the least affected dimensions were playing (0.9%) and schoolwork (1.2%). The main causes of impact were gingival bleeding (42.1%), sores (33.8%) and sensitivity (30.6%) and adolescents reported an average of 2.5 pathologies as a perceived cause of impact. Crooked teeth were the 4th cause of impact reported by the subjects.

A low intensity of impact was found in 54.6% of participants with impact, and a high impact intensity was found in 15% of cases, affecting psychosocial dimensions such as smiling (5.4% of the sample) and emotional state (2.7%). The extent of impact or Performances With Impact (PWI) was seven affected dimensions. Most of the participants with impact showed only one dimension affected (27% of the sample), while 12.2% reported two daily activities affected. Impact prevalence, extent of impact, the number of affected dimensions and the number of causes of impact showed a significant association with C-OIDP index (*p* < 0.05). Overall, the mean C-OIDP score was 3.28 and was mainly contributed to by the dimensions smiling (0.63), emotional state (0.51) and eating (0.47).

### 3.4. Clinical Exam

Dental caries was assessed using ICDAS II classification, and the DMFT index was calculated to allow for comparisons with other studies. The DMFT index was obtained from the ICDAS codes considering with respect to the caries extent codes 4 to 6 [28]. The percentage of participants with DMFT > 0 was 30.6%, and the majority had one or two teeth affected.

Regarding caries lesions, 65.6% of participants did not have incipient caries lesions (ICDAS caries codes 1 to 3); only 3.9% presented more than three incipient lesions. Moreover 87.8% of participants did not have advanced caries lesions (ICDAS caries codes 4 to 6), and only 1.8% presented ≥ 3 cavitated lesions. Data are shown in Table 4.

Regarding restorations, 78% of the sample did not have restorations, only 3% showed ≥3 restorations in good condition (ICDAS restoration code 3 to 6) and only two participants had one restoration in bad condition (ICDAS restoration code 7). Only three participants had one missing tooth due to caries and one participant two missed teeth due to caries (ICDAS code 9).

The DMFT index was 0.65, the weight of each component being D = 0.21, M = 0.01, and F = 0.42. The dental Morbidity Index (which refers to the relation between decayed teeth and DMFT index)(was 32.3%, the Restoration Index (which refers to the relation between filled teeth and DMFT index)(was 64.6% and the dental Mortality Index (which refers to the relation between missed teeth and DMFT index)was 1.5%. SiC (Significant Caries Index) was 1.94. Regarding pit and fissure sealants, 6.3% of participants had partially sealed teeth and 14.6% had well-sealed teeth.

The Community Periodontal Index (CPI) was 0.49, and 15% of participants had a CPI = 0. Table 5 shows the frequencies of distribution of CPI values for the index teeth. The maximum bleeding presence was at tooth 2.6, which was also the molar with the highest percentage of calcified plaque as well as the mandibular incisor group.

As far as occlusion is concerned, the data are shown in Table 6. Angle’s class was determined, and similar percentages were found for the prevalence of molar class. The most frequent alterations in occlusion were increased overjet and increased overbite, and 74.4% of the participants had no malocclusion in any of the three planes evaluated.

### 3.5. Association between the C-OIDP Index and the Clinical Variables

According to the findings of this study, the null hypothesis has been rejected.

The association between the C-OIDP index and the clinical variables is shown in Table 6; it can be noticed that the DMFT was found to be significantly associated with the C-OIDP index (*p* = 0.03). Spearman correlation between C-OIDP and DMF-T values was 0.12 (*p* = 0.02). Analysis by sex showed a significant association between quality of life and DMFT index for men (*p* = 0.03), but not for women (*p* = 0.50). The extent of caries lesions did not show significant association with the C-OIDP.

Although no significant association was found between CPI and the C-OIDP index, participants with CPI > 0 showed an impact index almost twice as high as those with CPI = 0 (Table 6). Analysis by sex demonstrated significant association between quality of life and CPI for women (*p* < 0.01), but not for men (*p* = 0.35).

The molar relationship evaluated by Angle’s class was associated with the impact index (*p* = 0.04). No other significant association was found between the evaluated occlusion variables and C-OIDP. However, participants with anterior open bite and posterior crossbite presented higher C-OIDP index, although without significant differences (*p* > 0.05) (Table 6). When men and women were analyzed independently, no significant differences were found.

## 4. Discussion

The C-OIDP questionnaire was chosen to assess oral quality of life. It has been validated in multiple cultural contexts [35,36,37,38] and shown adequate psychometric properties [19,20]. It allows impacts to be related to underlying pathologies causing them [16] and, by detecting the intensity and extent of the impact, has a greater discriminative ability that is useful for establishing treatment needs and measuring health outcomes [17,18]. The self-administered modality was selected, as self- and interviewer-administered C-OIDP have a high level of agreement [39].

Dental decay was assessed using ICDAS II classification. It has been encouraged in the last 2 decades to take caries evidence into clinical practice [40]. To our knowledge, this is the first time that ICDAS II has been used in studies of oral quality of life as measured with the C-OIDP index in adolescents. In most other studies, the WHO diagnostic criteria for caries were used, where a tooth is classified as decayed when a lesion in a pit or fissure or on a smooth or a proximal surface has the appearance of an unmistakable cavity or undermined enamel visible to the eye [25].

The DMFT index was obtained from the ICDAS codes considering with respect to the caries extent codes 4 to 6 [28].

The Community Periodontal Index (CPI) is recommended by WHO in oral health surveys [25] and was also used by other authors [41,42]. In other studies, gingival bleeding, visible plaque and O’Leary plaque indices were used [43,44].

Occlusal alterations were assessed by measuring discrepancies in the three planes of space [34]. Other indexes have been used, such as the Dental Aesthetic Index (DAI) [45,46,47] or Index of Orthodontic Treatment Needs (IOTN) [48].

This study showed a good internal reliability with a standardized Cronbach’s alpha of 0.69, very close to the value of 0.7 recommended by some authors. Alpha value depends on the correlation among the items in the scale and on the number of items; for scales with less than 10 items, lower values of Cronbach´s alpha can be expected. For purposes of group comparisons, a reliability of 0.5 or above is considered to be acceptable [49].

The prevalence of Impact of oral problems on daily living activities was moderate (48.1%), similar to Athira et al. (43%) [50], Yusuf et al. (40.4%) [37] and Dhawan et al. (49.4%) [51]. Previous studies carried out in urban settings reported higher prevalence of impact and in rural settings the results are inconclusive, ranging from Simangwa et al. (15%) [52] to Reinoso et al. (98%) [53], as compiled in the systematic review by Álvarez-Azaustre et al. [26]. The dimension most frequently affected was eating, and socializing (playing) was the least frequent, in agreement with previous studies [42,47,54,55,56].

The main cause of impact was gingival bleeding coinciding with Nordin et al. [57], while toothache was perceived as the first cause of impact in several studies [58,59]. The intensity of impact referred by adolescents was generally mild, in line with previous studies [59,60], and a high impact was only reported on psychosocial dimensions, such as smiling and emotional state, concurring with Nordin et al. [57], who also reported a high intensity on smiling.

The extent of impact was low, as most participants reported only one activity of daily living affected by oral problems, lower than that reported in previous studies, where the average number of affected dimensions varied between 3.9 and 4.8 depending on rural or urban setting, respectively, as summarized in a recent systematic review [26]. A mean C-OIDP = 3.28 was obtained, which can be considered low, similar to that reported by Arumrahayu et al. [61], and in the previous validation study in Spain [21]. Other authors found values ranging from 0.9 to 13.1 [62,63].

Regarding the relationship between dental caries and oral quality of life, a significant association was found between the DMFT index and the C-OIDP index, with a greater impact on quality of life in subjects with a higher DMFT index, coinciding with previous studies [46,63,64]. The existence of a DMFT index as low as 0.65 in our population may be the reason for not having found an association between the extent of the caries lesion and the impact index in our study. Nasia et al. [65] reported that children having at least one condition indicated by the PUFA index (Pulpal involvement, Ulceration, Fistula, Abscess) showed a much higher prevalence of oral impacts. In our study, none of the participants had PUFA conditions.

Periodontal status showed no significant association with the impact index, in agreement with reports by Bianco et al. [41] also using the CPI index and by other authors using the plaque index, gingival index or the O’Leary index; however, participants with CPI > 0 showed an impact index almost twice as high as those with CPI = 0. The presence of calculus and bleeding are the two aspects that are evaluated with the CPI index at this age. The occurrence of these two conditions is frequently associated with poor oral hygiene. Adolescents often lack established oral hygiene routines or their oral hygiene routines are ineffective in properly removing dental biofilm; this is associated with gingival swelling, bleeding or halitosis. All these conditions affect their quality of life. In this study, the perception of halitosis was reported by 34% of the participants and was significantly related to the impact index. The social impact of halitosis is profound for adolescents, harming their social life [66]. Likewise, the main cause of impact in the present study was gingival bleeding. Bakhtiar et al. [46] reported a significant relationship between the presence of dental plaque and C-OIDP scores. Recently, several authors reported that children with good oral hygiene (plaque index < 1) had significantly lower C-OIDP scores [61,65], and a gingival index > 1 was statistically associated with higher C-OIDP scores [61,63,64].

Regarding the occlusion alterations evaluated in the three planes of space in our study, only the Angle’s class showed a significant association with C-OIDP, being the participants with classes II and III those who had significantly higher mean values of C-OIDP. Although in our study the adolescents with anterior open bite and posterior crossbite showed a higher impact value, it did not reach statistical significance. Tooth position is an important aspect of the appearance and aesthetics of the smile. Therefore, smile and oral health can have an impact on daily life through social relationships and interactions of adolescents in the school environment.

Other authors have found an association between malocclusion and the C-OIDP index [41,47,50,63,64]. It has been reported that the best indicator of malocclusion to assess the impact on oral quality of life is the Dental Aesthetic Index (DAI), which captures the largest differences in impact index scores between adolescents with and without malocclusion [67]. This may be the reason why malocclusion, considered as binary occurrence (yes/no) had a low weight in the quality of life in this study.

The data obtained in our study regarding the clinical examination are similar to those of the last national Oral Health Survey in terms of caries prevalence, DMFT index, Restoration index and SiC (Significant Caries Index). The national survey reported 40.8% of the participants with CPI = 0 at 12 years of age and we found that 49% of the index teeth explored had a CPI = 0 [24]. Furthermore, the factors affecting oral quality of life in the sample analyzed are qualitatively similar to the sample selected in the national survey, so we consider that the external validity of the results can be established for the school population of the same age range at the national level.

The use of the C-OIDP questionnaire is useful for assessing health needs and treatment outcomes, including adolescent satisfaction with their oral health, providing valuable information for decision making, priority setting and resource allocation in dental care. Therefore, the use of this measurement tool in clinical and epidemiological practice is expected to increase in the coming years.

Among the limitations of the study is the cross-sectional design, so it is not possible to analyze the cause–effect relationship between the variables studied, but only the association between them. Test–retest reliability of the questionnaire could not be performed due to logistic reasons; therefore, the stability of the measurement instrument could not be checked, and the intraexaminer reliability could not be assessed due to the impossibility of screening, for a second time, a certain number of participants. Moreover, the Dental Aesthetic Index (DAI) may be a more appropriate index for detecting the association between malocclusion and oral quality of life, as each assessment method focuses on different aspects of occlusion that can be translated to specific domains of oral quality of life. Additionally, the Index of Orthodontic Treatment Needs-Aesthetic Component (IOTN-AC) points in the same direction in its relationship to oral quality of life [67].

A large proportion of adolescent health problems are lifestyle-related and therefore preventable. Lifestyles are also determinants of oral health. In accordance with the results of the present study, improving the oral health status of adolescents positively influences their oral quality of life. For this reason, public health programs and policies aimed at adolescents should not fail to include oral health aspects.

## 5. Conclusions

The OHRQoL in Spanish adolescents is good. The impact index was significantly higher in participants with a higher DMFT index, showing a greater impact in men than in women. Periodontal status had a greater impact in women than in men, while no differences by sex were found regarding the impact of malocclusion. No association between the extent of the caries lesion and the impact index was found.

## Figures and Tables

**Table 1:** ICDAS II (International Caries Detection and Assessment System) summary.

ICDAS II			
Restoration Code	Criteria	Caries Code	Criteria
0	No restoration or sealant	0	Healthy
1	Sealant in poor condition	1	Visual change in enamel after drying
2	Sealant in good condition	2	Visual change in enamel with wet tooth
3	Tooth-coloured filling	3	Localized enamel break
4	Amalgam	4	Underlying dark shade in dentine
5	Steel crown	5	Cavity with exposed dentine
6	Porcelain restoration	6	Extensive cavity
7	Lost or fractured filling		
8	Temporary restoration		

**Table 2:** Reliability analysis: Inter-item correlation for the C-OIDP.

C-OIDP	Eating	Speaking	Brushing	Sleeping	Emotion	Schoolwork	Smiling	Playing
Eating	1.00							
Speaking	0.28	1.00						
Brushing	0.16	0.47	1.00					
Sleeping	0.23	0.41	0.15	1.00				
Emotion	0.39	0.48	0.43	0.41	1.00			
Schoolwork	0.32	0.41	0.42	0.06	0.34	1.00		
Smiling	0.09	0.08	0.05	0.06	0.16	0.00	1.00	
Playing	0.15	0.13	0.09	0.08	0.07	0.07	0.00	1.000

**Table 3:** Reliability analysis: Corrected item-total correlations.

	Corrected Item-Total Correlation	Cronbach’s Alpha If Item is Deleted
Eating	0.38	0.59
Speaking	0.57	0.56
Brushing	0.40	0.59
Spleeping	0.36	0.60
Emotion	0.61	0.50
Schoolwork	0.39	0.62
Smiling	0.13	0.70
Playing	0.13	0.65

Cronbach’s alpha = 0.64. Standardized Cronbach’s alpha = 0.69.

**Table 4:** Participants with their ICDAS caries codes and number of teeth affected.

ICDAS 1–3			ICDAS 4–6		
N° of Teeth	*n*	%	N° of Teeth	*n*	%
0	221	65.6	0	296	87.8
1	62	18.4	1	25	7.4
2	29	8.6	2	10	3
3	12	3.6	3	4	1.2
4	8	2.4	4	1	0.3
5	3	0.9	10	1	0.3
6	1	0.3			
8	1	0.3			
Total	337	100	Total	337	100

**Table 5:** Community Periodontal Index (CPI) values for index teeth.

	CPI = 0 (Healthy)	CPI = 1 (Bleeding)	CPI = 2 (Calculus)
Index Teeth	% Participants		
1.6	49.6	40.7	9.8
1.1	60.5	35.3	4.2
2.6	37.4	50.4	12.2
4.6	53.7	43.3	3
3.1	46.3	16	37.7
3.6	49.3	47.8	3

**Table 6:** Association between clinical variables and C-OIDP index.

	C-OIDP					
Variables	*n*	Mean ± SD	95%CI	Median	Interq. Range	*p*
DMF-T Index = 0	234	3.16 ± 7.07	2.25–4.08	0	2.78	0.03
DMF-T Index > 0	103	3.53 ± 5.21	2.51–4.55	1.38	5.56	
ICDAS 1–3 = 0	221	3.45 ± 7.34	2.47–4.42	0	3.47	0.69
ICDAS 1–3 > 0	116	2.95 ± 4.71	2.09–3.82	1.38	4.17	
ICDAS 4–6 = 0	296	3.27 ± 6.74	2.50–4.04	0	2.78	0.89
ICDAS 4–6 > 0	41	3.31 ± 5.10	1.70–4.93	0	5.56	
CPI Index = 0	52	1.73 ± 2.47	1.04–2.42	0	2.78	0.36
CPI Index > 0	285	3.56 ± 7.01	2.74–4.38	0	4.17	
Malocclusion	86	3.1 ± 7.5	1.5–4.7	0	4.5	0.37
No malocclusion	251	3.3 ± 6.1	2.5–4.1	0	4.1	
No incisor crowding	249	3.37 ± 6.98	2.50–4.24	0	4.17	0.90
Incisor crowding	88	3.01 ± 5.17	1.91–4.11	0	2.78	
No incisor diastema	278	3.39 ± 6.89	2.58–4.21	0	4.17	0.88
Incisor diastema	59	2.73 ± 4.67	1.51–3.94	0	2.78	
No increased overbite	226	3.52 ± 7.30	2.57–4.48	0	4.17	0.68
Increased overbite	111	2.77 ± 4.66	1.89–3.65	0	4.17	
No increased overjet	219	3.19 ± 6.49	2.33–4.06	0	4.17	0.56
Increased overjet	118	3.43 ± 6.69	2.21–4.66	0	2.78	
No anterior open bite	315	3.21 ± 6.65	2.47–3.95	0	2.78	0.12
Anterior open bite	22	4.22 ± 4.93	2.04–6.41	0	8.33	
No posterior crossbite	310	3.19 ± 6.41	2.47–3.91	0	2.78	0.33
Posterior crossbite	27	4.28 ± 8.10	1.06–7.47	0	4.17	
Angle’s Class I	111	2.6 ± 5.1	1.6–3.6	0	2.7	0.04
Angle’s Class II	121	3.6 ± 5.2	2.7–4.6	1.3	5.5	
Angle’s Class III	105	3.4 ± 8.8	1.7–5.1	0	2.7	

## Data Availability

Data supporting results can be found at: https://www.educacion.gob.es/teseo (accessed on 11 August 2022).

## Supplementary Materials

The following are available online at https://www.mdpi.com/article/10.3390/ijerph191912450/s1, Figure S1: Child-OIDP questionnaire.

## Abbreviations

C-OIDP	Child-Oral Impacts on Daily Performances
CPI	Community Periodontal Index
CS-C-OIDP	Condition Specific Child-Oral Impacts on Daily Performances
DMFT	Decayed Missed Filled Tooth
ICDAS II	International Caries Detection and Assessment System
OHRQoL	Oral-Health-Related Quality of Life
QoL	Quality of Life
OSS	Oral Satisfaction Scale
SESPO	Sociedad Española de Epidemiología y Salud Pública Oral
WHO	World Health Organization.
