# Supplementary material for: Oral-Health-Related Quality of Life as Measured with the Child-OIDP Index and Oral Health Status in Spanish Adolescents
Source: Int J Environ Res Public Health. 2022 Sep 29;19(19):12450. doi: 10.3390/ijerph191912450 (PMC9564813; doi:10.3390/ijerph191912450)
Supplement: Supplementary file 1 [file ijerph-19-12450-s001.zip › ijerph-1932941-supplementary.pdf]

CHILD-OIDP QUESTIONNAIRE

Please answer the following questions and mark with a cross what is right:

1.- In the last three months, have you had problems eating (meals, ice creams,…) due to teeth pain?

- ☐ Yes
- ☐ No

If your answer is YES:
How has this problem affected your life?

☐ Almost nothing ☐ Something ☐ A Lot

How many times have you had this problem?

- ☐ Once or twice a month
- ☐ Three or more times a month, or once or twice a week
- ☐ Three or more per week

If you have had this problem eating less than once a month, during how many days occurred it in total?

- ☐ 1-7 days
- ☐ 8-15 days
- ☐ 15 days or more

Mark with a cross what you think has kept you from eating well in the last few months:

☐ Teeth pain	☐ Bleeding gums
☐ Teeth sensitivity	☐ Swelling of the gums
☐ Decayed teeth	☐ Tartar
☐ Baby teeth that have fallen out	☐ Mouth sores
☐ Hollows because the baby teeth did not come out	☐ Bad breath
	☐ Lump on the face
☐ Fractures of definitive teeth	☐ Emergence of teeth
☐ Changes in tooth color	☐ Some definitive tooth has fallen out
☐ Problems in size or shape	
☐ Crooked teeth	☐ Others

2.-Have you had problems speaking clearly and pronouncing well in these last three months due to problems in the mouth?

- ☐ Yes
- ☐ No

If your answer is YES:
How has this problem affected your life?

☐ Almost nothing ☐ Something ☐ A Lot

How many times have you had this problem?

- ☐ Once or twice a month
- ☐ Three or more times a month, or once or twice a week
- ☐ Three or more per week

If you have had this problem speaking less than once a month, during how many days occurred it in total?

- ☐ 1-7 days
- ☐ 8-15 days
- ☐ 15 days or more

Mark with a cross what you think has kept you from speaking well in the last few months:

☐ Teeth pain	☐ Bleeding gums
☐ Teeth sensitivity	☐ Swelling of the gums
☐ Decayed teeth	☐ Tartar
☐ Baby teeth that have fallen out	☐ Mouth sores
☐ Hollows because the baby teeth did not come out	☐ Bad breath
	☐ Lump on the face
☐ Fractures of definitive teeth	☐ Emergence of teeth
☐ Changes in tooth color	☐ Some definitive tooth has fallen out
☐ Problems in size or shape	
☐ Crooked teeth	☐ Others

3.-Have you been able to brush and rinse your teeth well during these last three months?

- ☐ Yes
- ☐ No

If your answer is YES:
How has this problem affected your life?

☐ Almost nothing ☐ Something ☐ A Lot

How many times have you had this problem?

- ☐ Once or twice a month
- ☐ Three or more times a month, or once or twice a week
- ☐ Three or more per week

5.- Have you been angry or annoyed in these last three months because your teeth hurt or you had a problem in your mouth?

- ☐ Yes
- ☐ No

If your answer is YES:
How has this problem affected your life?

☐ Almost nothing ☐ Something ☐ A Lot

How many times have you had this problem?

- ☐ Once or twice a month
- ☐ Three or more times a month, or once or twice a week
- ☐ Three or more per week

If you have had this problem in your mouth less than once a month, during how many days occurred it in total?

- ☐ 1-7 days
- ☐ 8-15 days
- ☐ 15 days or more

Mark with a cross what you think has kept you from eating well in the last few months:

☐ Teeth pain	☐ Bleeding gums
☐ Teeth sensitivity	☐ Swelling of the gums
☐ Decayed teeth	☐ Tartar
☐ Baby teeth that have fallen out	☐ Mouth sores
☐ Hollows because the baby teeth did not come out	☐ Bad breath
	☐ Lump on the face
☐ Fractures of definitive teeth	☐ Emergence of teeth
☐ Changes in tooth color	☐ Some definitive tooth has fallen out
☐ Problems in size or shape	
☐ Crooked teeth	☐ Others

6.- Have you been embarrassed to smile or show your teeth in these last three months because you do not like them?

- ☐ Yes
- ☐ No

If your answer is YES:
How has this problem affected your life?

☐ Almost nothing ☐ Something ☐ A Lot

How many times have you had this problem?

- ☐ Once or twice a month
- ☐ Three or more times a month, or once or twice a week
- ☐ Three or more per week

If you have had this problem smiling less than once a month, during how many days occurred it in total?

- ☐ 1-7 days
- ☐ 8-15 days
- ☐ 15 days or more

Mark with a cross what you think has kept you from smiling and showing your teeth in the last few months:

☐ Teeth pain	☐ Bleeding gums
☐ Teeth sensitivity	☐ Swelling of the gums
☐ Decayed teeth	☐ Tartar
☐ Baby teeth that have fallen out	☐ Mouth sores
☐ Hollows because the baby teeth did not come out	☐ Bad breath
	☐ Lump on the face
☐ Fractures of definitive teeth	☐ Emergence of teeth
☐ Changes in tooth color	☐ Some definitive tooth has fallen out
☐ Problems in size or shape	
☐ Crooked teeth	☐ Others

7.- Have you ever had to stop doing homework or housework, or not go to school because your teeth hurt in these last three months?

- ☐ Yes
- ☐ No

If your answer is YES:
How has this problem affected your life?

☐ Almost nothing ☐ Something ☐ A Lot

How many times have you had this problem?

- ☐ Once or twice a month
- ☐ Three or more times a month, or once or twice a week
- ☐ Three or more per week

If you have had this problem to brush your teeth less than once a month, during how many days occurred it in total?

- ☐ 1-7 days
- ☐ 8-15 days
- ☐ 15 days or more

Mark with a cross what you think has kept you from brushing your teeth well in the last few months:

☐ Teeth pain	☐ Bleeding gums
☐ Teeth sensitivity	☐ Swelling of the gums
☐ Decayed teeth	☐ Tartar
☐ Baby teeth that have fallen out	☐ Mouth sores
☐ Hollows because the baby teeth did not come out	☐ Bad breath
	☐ Lump on the face
☐ Fractures of definitive teeth	☐ Emergence of teeth
☐ Changes in tooth color	☐ Some definitive tooth has fallen out
☐ Problems in size or shape	
☐ Crooked teeth	☐ Others

If you have had this problem to do homework less than once a month, during how many days occurred it in total?

- ☐ 1-7 days
- ☐ 8-15 days
- ☐ 15 days or more

Mark with a cross what you think has kept you from doing homework or going to school in the last few months:

☐ Teeth pain	☐ Bleeding gums
☐ Teeth sensitivity	☐ Swelling of the gums
☐ Decayed teeth	☐ Tartar
☐ Baby teeth that have fallen out	☐ Mouth sores
☐ Hollows because the baby teeth did not come out	☐ Bad breath
	☐ Lump on the face
☐ Fractures of definitive teeth	☐ Emergence of teeth
☐ Changes in tooth color	☐ Some definitive tooth has fallen out
☐ Problems in size or shape	
☐ Crooked teeth	☐ Others

4.-Have you had pain or any problems in your mouth in these last three months that have prevented you from resting or sleeping well?

- ☐ Yes
- ☐ No

If your answer is YES:

How has this problem affected your life?

☐ Almost nothing ☐ Something ☐ A Lot

How many times have you had this problem?

- ☐ Once or twice a month
- ☐ Three or more times a month, or once or twice a week
- ☐ Three or more per week

If you have had this problem in your mouth less than once a month, during how many days occurred it in total?

- ☐ 1-7 days
- ☐ 8-15 days
- ☐ 15 days or more

Mark with a cross what you think has kept you from resting or sleeping well in the last few months:

☐ Teeth pain	☐ Bleeding gums
☐ Teeth sensitivity	☐ Swelling of the gums
☐ Decayed teeth	☐ Tartar
☐ Baby teeth that have fallen out	☐ Mouth sores
☐ Hollows because the baby teeth did not come out	☐ Bad breath
	☐ Lump on the face
☐ Fractures of definitive teeth	☐ Emergence of teeth
☐ Changes in tooth color	☐ Some definitive tooth has fallen out
☐ Problems in size or shape	
☐ Crooked teeth	☐ Others

8.- In these last three months, have you ever not been able to go and play with your friends because your teeth hurt?

- ☐ Yes
- ☐ No

If your answer is YES:

How has this problem affected your life?

☐ Almost nothing ☐ Something ☐ A Lot

How many times have you had this problem?

- ☐ Once or twice a month
- ☐ Three or more times a month, or once or twice a week
- ☐ Three or more per week

If you have had this problem to play less than once a month, during how many days occurred it in total?

- ☐ 1-7 days
- ☐ 8-15 days
- ☐ 15 days or more

Mark with a cross what you think has kept you from going and play with your friends in the last few months:

☐ Teeth pain	☐ Bleeding gums
☐ Teeth sensitivity	☐ Swelling of the gums
☐ Decayed teeth	☐ Tartar
☐ Baby teeth that have fallen out	☐ Mouth sores
☐ Hollows because the baby teeth did not come out	☐ Bad breath
	☐ Lump on the face
☐ Fractures of definitive teeth	☐ Emergence of teeth
☐ Changes in tooth color	☐ Some definitive tooth has fallen out
☐ Problems in size or shape	
☐ Crooked teeth	☐ Others

Manuscript ID: ijerph-1932941

SUPPLEMENTARY MATERIALS:

FIGURE S1: CHILD-ORAL IMPACTS ON DAILY PERFORMANCES questionnaire
